# *APC* gene mutations and colorectal adenomatosis in familial adenomatous polyposis

**DOI:** 10.1054/bjoc.1999.0925

**Published:** 2000-01-18

**Authors:** F Ficari, A Cama, R Valanzano, M C Curia, R Palmirotta, G Aceto, D L Esposito, S Crognale, A Lombardi, L Messerini, R Mariani-Costantini, F Tonelli, P Battista

**Affiliations:** Unit of Surgery, Department of Clinical Physiopathology and Institute of Anatomic Pathology, University of Florence, Viale Morgagni 85, Florence, 50134, Italy; Section of Molecular Pathology, Department of Oncology and Neurosciences, University Gabriele D'Annunzio, Via dei Vestini 1, 66013 Chieti, Italy

**Keywords:** FAP (familial adenomatous polyposis), *APC* (adenomatous polyposis coli) gene, germline mutations, colorectal adenomas, number, distribution

## Abstract

Correlations between germline *APC* mutation sites and colorectal pathophenotypes, as evaluated by the direct count of adenomas at colectomy, were investigated analysing colectomy specimens from 29 FAP patients carrying one mis-sense (codon 208) and 14 frame-shift or non-sense *APC* mutations (codons 232, 367, 437, 623, 876, 995, 1061, 1068, 1075, 1112, 1114, 1309, 1324, 1556). The mis-sense mutation at codon 208 was associated with a relatively mild colorectal pathophenotype. The mutation at codon 367, subject to alternative splicing, was associated with attenuated FAP. The mutation at codon 1309 was associated with the profuse colorectal adenomatosis. For 13 mutations, predicted to result in null alleles or truncated APC proteins, we correlated density and distribution of colorectal adenomas with the predicted functional effects of the mutation. The most severe colorectal pathophenotype was significantly associated with the truncating mutation at codon 1309, which is located downstream to the I β-catenin binding domain but upstream II β-catenin-binding domain. Mutations between codons 867 and 1114, which affect the I β-catenin binding domain, as well as mutations occurring in exons 6 and 9, predicted to result in null alleles, were associated with a less severe colorectal pathophenotype. Overall, the highest number of adenomas was detected in the right colon, followed by the left colon, transverse colon sigma and rectum. However, the highest density of adenomas was observed in the left colon, followed by the right colon, sigma, transverse colon and rectum. Colorectal carcinomas, observed in only five patients, were all in the left colon. © 2000 Cancer Research Campaign


*British Journal of Cancer* (2000) **82**(2), 348–353
© 2000 Cancer Research Campaign
Article no. bjoc.1999.0925

### *APC* gene mutations and colorectal adenomatosis in familial adenomatous polyposis

**F Ficari[1], A Cama[3], R Valanzano[1], MC Curia[3], R Palmirotta[3], G Aceto[3], DL Esposito[3], S Crognale[3], A Lombardi[1], L Messerini[2], R Mariani-Costantini[3], F Tonelli[1] and P Battista[3]**

[1]Unit of Surgery, Department of Clinical Physiopathology and [2]Institute of Anatomic Pathology, University of Florence, Viale Morgagni, 85, 50134 Florence, Italy; [3]Section of Molecular Pathology, Department of Oncology and Neurosciences, University Gabriele D'Annunzio, Via dei Vestini 1, 66013 Chieti, Italy

**Summary** Correlations between germline *APC* mutation sites and colorectal pathophenotypes, as evaluated by the direct count of adenomas at colectomy, were investigated analysing colectomy specimens from 29 FAP patients carrying one mis-sense (codon 208) and 14 frame-shift or non-sense *APC* mutations (codons 232, 367, 437, 623, 876, 995, 1061, 1068, 1075, 1112, 1114, 1309, 1324, 1556). The mis-sense mutation at codon 208 was associated with a relatively mild colorectal pathophenotype. The mutation at codon 367, subject to alternative splicing, was associated with attenuated FAP. The mutation at codon 1309 was associated with the profuse colorectal adenomatosis. For 13 mutations, predicted to result in null alleles or truncated APC proteins, we correlated density and distribution of colorectal adenomas with the predicted functional effects of the mutation. The most severe colorectal pathophenotype was significantly associated with the truncating mutation at codon 1309, which is located downstream to the I β-catenin binding domain but upstream II β-catenin-binding domain. Mutations between codons 867 and 1114, which affect the I β-catenin binding domain, as well as mutations occurring in exons 6 and 9, predicted to result in null alleles, were associated with a less severe colorectal pathophenotype. Overall, the highest number of adenomas was detected in the right colon, followed by the left colon, transverse colon sigma and rectum. However, the highest density of adenomas was observed in the left colon, followed by the right colon, sigma, transverse colon and rectum. Colorectal carcinomas, observed in only five patients, were all in the left colon. © 2000 Cancer Research Campaign

**Keywords:** FAP (familial adenomatous polyposis); *APC* (adenomatous polyposis coli) gene; germline mutations; colorectal adenomas; number; distribution

Familial adenomatous polyposis (FAP) is an autosomal dominant inherited disease due to mutations in the tumour suppressor adenomatous polyposis coli (*APC*) gene (Bodmer et al, 1987; Groden et al, 1991; Kinzler et al, 1991). FAP is characterized by the development of multiple adenomatous polyps throughout the large intestine (Haggitt et al, 1986; Utsunomiya, 1989) and is associated with a very high risk of colorectal cancer and with a variety of extra-colorectal disease manifestations (Jones et al, 1986; Jagelman, 1987; Baker et al, 1988; Morton et al, 1992; Olschwang et al, 1993; Caspari et al, 1995; Valanzano et al, 1996). The *APC* gene is comprised of 15 exons, of which exon 15 encodes for more than 50% of the protein (Groden et al, 1991; Kinzler et al, 1991). The APC protein is composed of 2843 amino acids and mediates growth regulatory signals by its association with the microtubule cytoskeleton and with the cadherin-binding protein β-catenin. Amino-terminal residues 1–171 are implicated in homodimerization, being the first 55 amino acids sufficient to form a stable dimer (Joslyn et al, 1993). Amino-terminal residues 453–767 contain seven copies of a repeating 42 amino acids motif, originally identified in the *Drosophila* segment polarity gene product armadillo, a component of the wingless transduction pathway (Peifer et al, 1994). Furthermore, the APC protein binds β-catenin through two motifs: the first, located between residues 1020–1169, which contains three 15 amino acid repeats (Rubinfeld et al, 1993; Su et al, 1993); the second, located between residues 1342–2075, which comprises 7 repeats of 20 amino acids (Rubilfeld et al, 1997). This second motif acts also as substrate for serine-threonine glycogen synthase kinase-3β (GSK-3β) association and phosphorylation (Rubinfeld et al, 1996). The large body of data collected on *APC* mutations in FAP has contributed to establish correlations between type and location of the mutation and clinicopathologic features of the disease. There is a wide variability in the clinical presentation of FAP, in terms of age at disease onset and number and distribution of colorectal adenomatous polyps (Giardiello et al, 1994; Presciuttini et al, 1994). Certain phenotypic characteristics of FAP appear to be correlated with the site of *APC* mutations (Spirio et al, 1993; Caspari et al, 1994; Gayther et al, 1994; Friedl et al, 1996; Soravia et al, 1998). Mutations at the very 5′ end (codons 1–157) and towards the 3′ end of the gene (i.e. beyond codon 1578) are associated with a form of FAP characterized by relatively low numbers of colorectal adenomatous polyps (5–100) and comparatively late disease onset, designated attenuated adenomatous polyposis coli (AAPC) (Leppert et al, 1990; Friedl et al, 1996; Soravia et al, 1998). Furthermore, we and others described attenuated forms of FAP in kindreds with germline mutations in regions of the *APC* gene that are subjected to alternative splicing (Curia et al, 1998). On the contrary, the most frequent *APC* germline mutation, localized at codon 1309, is associated with a very severe form of FAP, characterized by hundreds to thousands colorectal adenomas and early onset (Caspari et al, 1994; Gayther

*Received 1 December 1998*
*Revised 22 June 1999*
*Accepted 3 August 1999*

*Correspondence to*: A Cama et al, 1994; Cama et al, 1995). Untreated patients with the codon 1309 *APC* gene mutation die on the average 10 years earlier than FAP patients with germline mutations at sites other than codon 1309 (Caspari et al, 1994; Gayther et al, 1994). Interestingly, codon 1309 lies within the 'mutation cluster region' (codons 1286–1514), i.e. the region of the *APC* gene that exhibits a definite accumulation of somatic mutations in both FAP-associated and non-FAP-associated colorectal tumours (Miyoshi et al, 1992; Miyaki et al, 1994; Yashima et al, 1994).

In spite of a number of important studies that documented the genetic bases of phenotypic heterogeneity in FAP (Leppert et al, 1990; Morton et al, 1992; Caspari et al, 1994; Gayther et al, 1994; Giardiello et al, 1994; Friedl et al, 1996; Soravia et al, 1998), there is a scarcity of data concerning correlations between number of colorectal adenomas, as evaluated by direct count on colectomy specimens, and position of the germline *APC* gene mutation responsible for the disease. The present study correlates colorectal disease manifestations with the results of *APC* mutational analysis in 29 colectomized FAP patients from 21 unrelated kindreds.

### MATERIALS AND METHODS

### Patients

Twenty-nine FAP patients from 21 unrelated kindreds were enroled in the study. All patients were treated with colectomy. The age at colectomy depended solely on the extent of the disease progression and ranged from 10 to 62 years (average 24.7). For each case, the area of mucosal surface of each colorectal segment (right, transverse and left colon, sigma and rectum), as well as the total area of colorectal mucosal surface, were obtained based on measurements taken on fresh colectomy specimens. The number of adenomas in the right, transverse and left colon, sigma and rectum was determined after the careful count of polyps visible on the mucosal surface of the fresh colectomy specimen and randomly confirmed by microscopic examinations. The density of adenomas per 4 $cm^2$ unit area of mucosal surface was calculated by dividing the number of adenomas determined in each anatomical segment, or in the entire colorectum, by the respective area of mucosal surface, expressed in $cm^2$. In kindred GD-11, because of the profuse adenomatosis, the number of adenomas was extrapolated by counts limited to representative 4 $cm^2$ unit areas of mucosal surface for each anatomical segment of the colorectum.

### Mutational analysis of the APC gene

Mutational analysis of the *APC* gene was conducted after full informed consent, using genomic DNA and/or total RNA extracted from fresh peripheral blood lymphocytes. Genetic analyses were always confirmed using DNA deriving from two independent blood samples and were performed employing a combination of three screening strategies, followed by direct sequencing (Cama et al, 1991). Heteroduplex analysis on agarose minigel (HAAM), coupled with allele-specific multiplex PCR, was designed to rapidly identify the three frequently occurring deletions of the *APC* gene at codons 1061, 1068 and 1309 (Cama et al, 1995). Single-strand conformation polymorphism (SSCP) analysis was used to screen the coding sequence of the *APC* gene spanning exons 1 through 14 (Groden et al, 1991, 1993; Cama et al, 1994). The in vitro synthesized protein (IVSP) assay was used for the rapid identification of truncating mutations in exon 15 (van der Luijt et al, 1994; Valanzano et al, 1996). Primers used for amplifications were as reported elsewhere (Groden et al, 1991, 1993; Miyoshi et al, 1992; van der Luijt et al, 1994). The research protocol was approved by the ethical review board of the University Gabriele D'Annunzio.

### Statistical analysis

The total number of adenomas at colectomy, the density of adenomas per unit area of mucosal surface obtained for the entire colorectum and for each colorectal anatomical segment were correlated with age at colectomy and with site of *APC* mutation. Statistical evaluations were performed using the analysis of variance (ANOVA-test). Probability values (*P*-values) of less than 0.05 were considered significant.

### RESULTS

The combined use of the HAAM, SSCP and IVSP assays for mutational analysis allowed the detection of 15 different germline *APC* mutations responsible for disease in 29 FAP patients from 21 unrelated kindreds (Table 1 and Figure 1). Ten mutations were clustered at the 5′ end of exon 15, between codons 876 and 1556. Five mutations were respectively located within exons 5 (codon 208), 6 (codon 232), 9 (codons 367 and 437), and 14 (codon 623). With the exception of the novel mis-sense mutation at codon 208, all other mutations are predicted to introduce premature termination signals. The mutation at codon 1324 determines a frame-shift introducing a distant stop signal 90 codons downstream. The mis-sense mutation at codon 208 is considered pathogenic on the basis of its co-segregation with 3 FAP patients in the kindred (information kindly supplied by Dr L Varesco, Istituto Nazionale Ricerca Cancro, Genova, Italy and confirmed in our laboratory) and lack of segregation in unaffected members of the family. This APC allelic variant is unlikely to be a common polymorphism as it has not been detected in additional 118 chromosomes screened.

Clinicopathological parameters, including age at colectomy, total number and anatomical distribution of colorectal adenomas, density of adenomas per unit area of mucosal surface were correlated with *APC* mutation sites. The total number of colorectal adenomas detected on fresh at colectomy specimens appeared to vary among FAP patients within the same family, as exemplified in kindreds GD-1, GD-3, GD-4, and GD-15 (Figure 1). These differences appeared to be independent from age at colectomy. Variability in the total number of adenomas was also observed among FAP patients from unrelated families carrying the same *APC* mutation (Figure 1). In spite of this variability, carriers of the codon 1309 mutation manifested a significantly higher number of adenomas at colectomy (from 450 to 7460), compared to carriers of other APC mutations (from 22 to 1750) ($P < 0.01$). There was a significant anticipation in the age at colectomy in carriers of mutation at codon 1309 (average 16.6; s.e.m. ± 2.9) versus carriers of other *APC* mutations (average 28.5; s.e.m. ± 2.8) ($P < 0.01$). Six out of the ten codon 1309 mutation carriers underwent colectomy during the 2nd decade of life (Figure 1).

Colorectal cancers occurred in five patients, respectively carrying mutations at codon 876 (one patient), 1068 (two patients) and 1309 (two patients) (Figure 1). It should be noted that only one patient had cancer before age 30 years and that this patient carried

© 2000 Cancer Research Campaign

**Table 1** Summary of the germline mutations of the APC gene detected in 21 FAP kindreds

| Kindred | Mutation | Codon | Type of mutation | Exon |
|---|---|---|---|---|
| GD-16 | CCA → CCG | 208 | Gln → Arg | 5 |
| GD-21 | CGA → TGA | 232 | Arg → Stop | 6 |
| GD-17 | GAC TCT → GAC T | 367 | 2 bp deletion | 9 |
| GD-2 | AAT CC*A A/g*tatg → AAT CC /tatg | 437 | 3 bp deletion | 9 (donor site) |
| GD-24 | TAC CGG → TAC TTAC CGG | 623 | 4 bp insertion | 14 |
| GD-18 | CGA → TGA | 876 | Arg → Stop | 15 |
| GD-19 | TGC → TGA | 995 | Cys → Stop | 15 |
| GD-8 | AA ATA AAA CAA AGT → AA AT AA AGT | 1061 | 5 bp deletion | 15 |
| GD-9 | AA ATA AAA CAA AGT → AA AT AA AGT | 1061 | 5 bp deletion | 15 |
| GD-4 | AGA CAA TCA AGG A → AGA CA AGG A | 1068 | 4 bp deletion | 15 |
| GD-13 | TAT → TAA | 1075 | Tyr → Stop | 15 |
| GD-12 | GAA *A*CA AAT → GAA CA AAT | 1112 | 1 bp deletion | 15 |
| GD-15 | CGA → TGA | 1114 | Arg → Stop | 15 |
| GD-3 | AAA GAA AAG ATT → AAA GA TT | 1309 | 5 bp deletion | 15 |
| GD-5 | AAA GAA AAG ATT → AAA GA TT | 1309 | 5 bp deletion | 15 |
| GD-6 | AAA GAA AAG ATT → AAA GA TT | 1309 | 5 bp deletion | 15 |
| GD-7 | AAA GAA AAG ATT → AAA GA TT | 1309 | 5 bp deletion | 15 |
| GD-10 | AAA GAA AAG ATT → AAA GA TT | 1309 | 5 bp deletion | 15 |
| GD-11 | AAA GA*A* AAG ATT → AAA GA TT | 1309 | 5 bp deletion | 15 |
| GD-1 | CCA GCA → CA GCA | 1324 | 1 bp deletion | 15 |
| GD-27 | GCA GAA → GCA A GAA | 1556 | 1 bp insertion | 15 |

HAAM, coupled with allele-specific multiplex PCR allowed to detect mutations at codons 1061, 1068 and 1309. SSCP allowed to detect mutations at codons 208, 232, 367, 437, 623, 1075 and 1324 and IVSP allowed to detect mutations at codons 876, 995, 1112, 1114 and 1556.

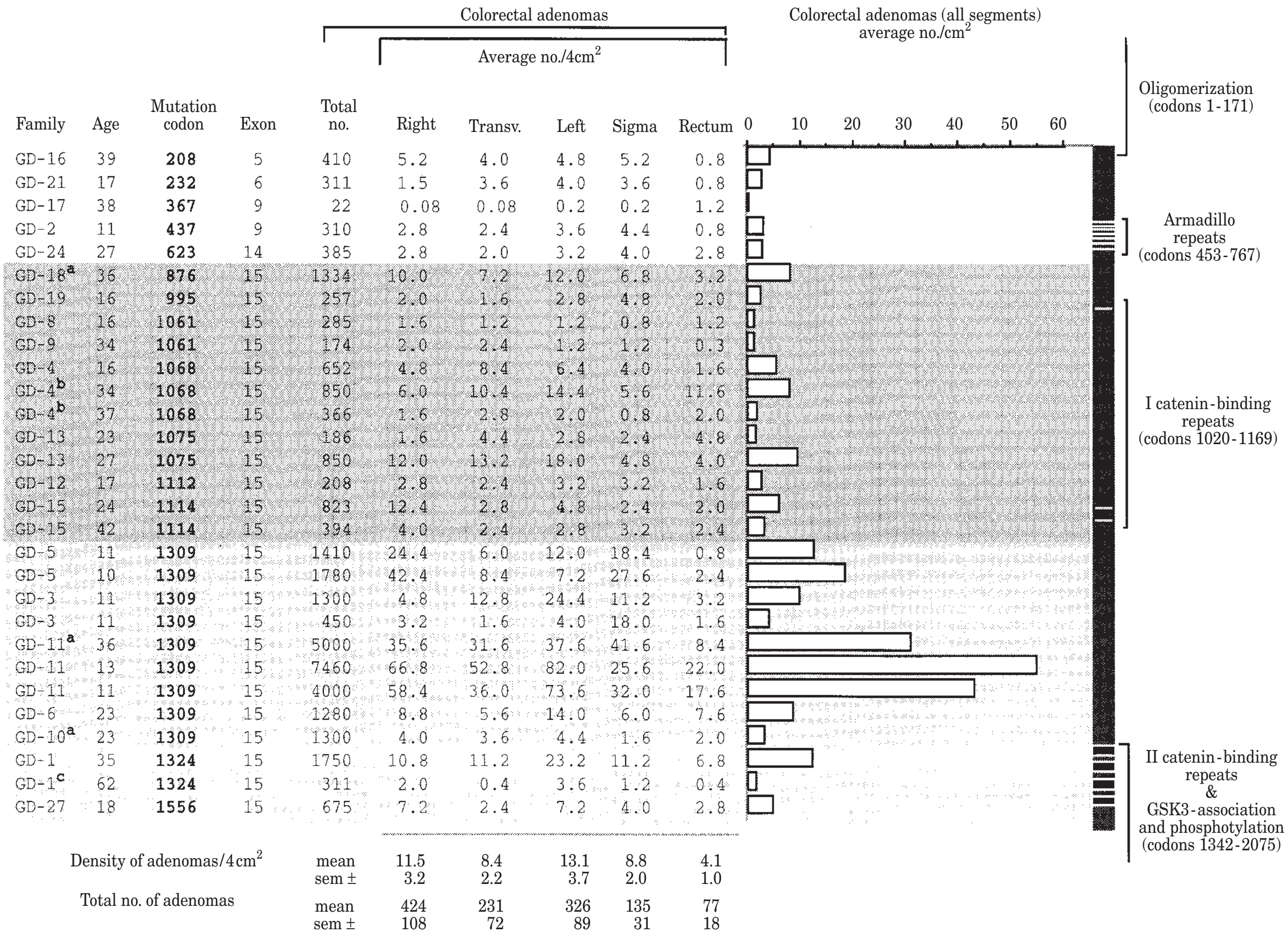

| Family | Age | Mutation codon | Exon | Total no. | Right | Transv. | Left | Sigma | Rectum |
|---|---|---|---|---|---|---|---|---|---|
| GD-16 | 39 | **208** | 5 | 410 | 5.2 | 4.0 | 4.8 | 5.2 | 0.8 |
| GD-21 | 17 | **232** | 6 | 311 | 1.5 | 3.6 | 4.0 | 3.6 | 0.8 |
| GD-17 | 38 | **367** | 9 | 22 | 0.08 | 0.08 | 0.2 | 0.2 | 1.2 |
| GD-2 | 11 | **437** | 9 | 310 | 2.8 | 2.4 | 3.6 | 4.4 | 0.8 |
| GD-24 | 27 | **623** | 14 | 385 | 2.8 | 2.0 | 3.2 | 4.0 | 2.8 |
| GD-18[a] | 36 | **876** | 15 | 1334 | 10.0 | 7.2 | 12.0 | 6.8 | 3.2 |
| GD-19 | 16 | **995** | 15 | 257 | 2.0 | 1.6 | 2.8 | 4.8 | 2.0 |
| GD-8 | 16 | **1061** | 15 | 285 | 1.6 | 1.2 | 1.2 | 0.8 | 1.2 |
| GD-9 | 34 | **1061** | 15 | 174 | 2.0 | 2.4 | 1.2 | 1.2 | 0.3 |
| GD-4 | 16 | **1068** | 15 | 652 | 4.8 | 8.4 | 6.4 | 4.0 | 1.6 |
| GD-4[b] | 34 | **1068** | 15 | 850 | 6.0 | 10.4 | 14.4 | 5.6 | 11.6 |
| GD-4[b] | 37 | **1068** | 15 | 366 | 1.6 | 2.8 | 2.0 | 0.8 | 2.0 |
| GD-13 | 23 | **1075** | 15 | 186 | 1.6 | 4.4 | 2.8 | 2.4 | 4.8 |
| GD-13 | 27 | **1075** | 15 | 850 | 12.0 | 13.2 | 18.0 | 4.8 | 4.0 |
| GD-12 | 17 | **1112** | 15 | 208 | 2.8 | 2.4 | 3.2 | 3.2 | 1.6 |
| GD-15 | 24 | **1114** | 15 | 823 | 12.4 | 2.8 | 4.8 | 2.4 | 2.0 |
| GD-15 | 42 | **1114** | 15 | 394 | 4.0 | 2.4 | 2.8 | 3.2 | 2.4 |
| GD-5 | 11 | **1309** | 15 | 1410 | 24.4 | 6.0 | 12.0 | 18.4 | 0.8 |
| GD-5 | 10 | **1309** | 15 | 1780 | 42.4 | 8.4 | 7.2 | 27.6 | 2.4 |
| GD-3 | 11 | **1309** | 15 | 1300 | 4.8 | 12.8 | 24.4 | 11.2 | 3.2 |
| GD-3 | 11 | **1309** | 15 | 450 | 3.2 | 1.6 | 4.0 | 18.0 | 1.6 |
| GD-11[a] | 36 | **1309** | 15 | 5000 | 35.6 | 31.6 | 37.6 | 41.6 | 8.4 |
| GD-11 | 13 | **1309** | 15 | 7460 | 66.8 | 52.8 | 82.0 | 25.6 | 22.0 |
| GD-11 | 11 | **1309** | 15 | 4000 | 58.4 | 36.0 | 73.6 | 32.0 | 17.6 |
| GD-6 | 23 | **1309** | 15 | 1280 | 8.8 | 5.6 | 14.0 | 6.0 | 7.6 |
| GD-10[a] | 23 | **1309** | 15 | 1300 | 4.0 | 3.6 | 4.4 | 1.6 | 2.0 |
| GD-1 | 35 | **1324** | 15 | 1750 | 10.8 | 11.2 | 23.2 | 11.2 | 6.8 |
| GD-1[c] | 62 | **1324** | 15 | 311 | 2.0 | 0.4 | 3.6 | 1.2 | 0.4 |
| GD-27 | 18 | **1556** | 15 | 675 | 7.2 | 2.4 | 7.2 | 4.0 | 2.8 |
| Density of adenomas/4cm² | | | | mean | 11.5 | 8.4 | 13.1 | 8.8 | 4.1 |
| | | | | sem ± | 3.2 | 2.2 | 3.7 | 2.0 | 1.0 |
| Total no. of adenomas | | | | mean | 424 | 231 | 326 | 135 | 77 |
| | | | | sem ± | 108 | 72 | 89 | 31 | 18 |

**Figure 1** Age at colectomy, site and exon of *APC* gene mutations, total number of colorectal adenomas and average number of adenomas per unit area of mucosal surface in the colorectal anatomical segments. The histogram illustrates the average number of adenomas per unit area of colorectal mucosa, as evaluated for the entire colorectum in each patient. A schematic representation of the APC functional domains affected by the mutations is shown to the right (not in scale). (a), patients affected by invasive cancer; (b), patients affected by 'in situ' colorectal cancer; (c) patients affected by duodenal cancer at 62 years of age

*© 2000 Cancer Research Campaign*

the codon 1309 mutation. A carrier of the codon 1324 mutation, affected with duodenal carcinoma, was still negative for colorectal cancer in the seventh decade.

Considering all the 29 patients and regardless of the relative areas of mucosal surface of each colonic segment, the highest number of adenomas was localized in the right colon, followed by the left and transverse colon, sigma and rectum (Figure 1). However, the density of adenomas per unit area of mucosal surface (4 $cm^2$) was highest in the left colon, followed by the right colon, sigma, transverse and rectum (Figure 1).

To investigate possible correlations between the colorectal pathophenotypes and the predicted effects of the mutations on the APC protein, we focused on frame-shift and splice-site mutations. The novel mis-sense mutation at codon 208 and the previously described mutation at codon 367, that was associated to a transcript dosage effect resulting in an attenuated phenotype (Curia et al, 1998), were not considered for these specific correlations. The 27 FAP patients carrying truncating mutations were divided into three subsets (Figures 1 and 2). Subset no. 1 included patients with mutations at codons 232, 437 and 623, proven or predicted to result in lack of expression of the mutant alleles (Smith et al, 1993; Curia et al, 1998). Subset no. 2 carried mutations at codons 876, 995, 1061, 1068, 1075, 1112 and 1114, predicted to result in APC proteins truncated downstream to the armadillo repeats but upstream to or within the I β-catenin-binding repeats. Subset no. 3 carried mutations at codons 1309, 1324 and 1556, predicted to result in APC proteins truncated downstream to the I β-catenin-binding repeats but upstream to or within the II β-catenin-binding repeats and GSK-3β association and phosphorylation sites. The three subsets of patients demonstrated differences in the mean number of total colorectal adenomas, that corresponded to 335 (s.e.m. ± 24) for subset no. 1, to 531 (s.e.m. ± 106) for subset no. 2, and to 2226 (s.e.m. ± 623) for subset no. 3 ($P = 0.009$), as well as in the average number of adenomas per units of area of colorectal mucosa (Figure 2). Subset no. 2 tended to associate with higher number and density of adenomas than subset no. 1, but the differences between these subsets were not statistical. The most severe colorectal pathophenotype, in terms of total number and density of adenomas per unit area of mucosal surface, was associated with subset no. 3 (Figure 2).

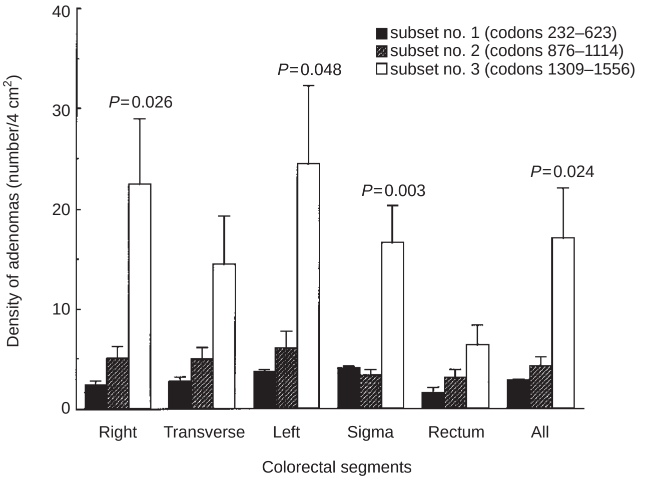

**Figure 2** Average number of colorectal adenomas in the three subsets of FAP patients with *APC* mutations predicted to result in truncated proteins or null alleles. Mutations at codons 208 and 367 are not considered in this histogram. Probability values (*P*) calculated by analysis of variance (ANOVA-test)

### DISCUSSION

The vast majority of FAP patients in our series carried mutations in exon 15 and the most frequent was the 5-bp deletion at codon 1309, which is in agreement with other studies (Nagase et al, 1992; Gayther et al, 1994). Ten of the 15 *APC* mutations discussed in the present study are predicted to result in the expression of proteins truncated at various levels in the carboxyterminal domain, that contains important growth suppressor activities (Nagase et al, 1993; Smith et al, 1993; Munemitsu et al, 1994; Matsumine et al, 1996). Four of the 15 mutations occurring between codons 232–623 are predicted to result in null alleles (Smith et al, 1993; Curia et al, 1998). Among these, the mutation at codon 367 in exon 9 is attenuated because of differential splicing (Curia et al, 1998) and was associated with a colorectal pathophenotype within the clinicopathological spectrum of AAPC (Leppert et al, 1990; Soravia et al, 1998). Finally, one of the 15 mutations resulted in the replacement of a negatively-charged with a positively-charged amino acid residue at codon 208. The functional implications of this mutation are unclear, although the mis-sense variant was associated with a relatively mild colorectal pathophenotype.

A crucial function of the multidomain APC protein is that of cooperating with GSK-3β in the regulation of free β-catenin level (Dietrich et al, 1997; Morin et al, 1997; Sparks et al, 1998). The association of β-catenin with its binding sites in the APC protein and the phosphorylation of APC by GSK-3β are prerequisites for β-catenin down-regulation. Free cytoplasmic β-catenin and Tcf family proteins are implicated in the constitutive activation of a signalling pathway promoting cell growth and differentiation, which is suspected to cause adenoma formation following *APC* gene mutation (Dietrich et al, 1997; Morin et al, 1997; Sparks et al, 1998). For 13 mutations predicted to result in null alleles or truncated APC proteins we correlated number and distribution of colorectal adenomas with the predicted functional effects of the *APC* mutations. In our series, the density of colorectal adenomas was not statistically different between mutations predicted to result in a null allele and mutations predicted to truncate the protein before or within the I β-catenin binding repeats. Conversely, mutations predicted to result in APC proteins truncated downstream to the I β-catenin-binding site but upstream to or within the II β-catenin-binding repeats and GSK-3β association and phosphorylation sites were associated with a more aggressive phenotype in terms of density of colorectal adenomas. As in other studies (Caspari et al, 1994; Gayther et al, 1994), the most aggressive colorectal pathophenotype, both in terms of overall number of colorectal adenomas and of age at colectomy, was associated with the truncating mutation at codon 1309, which is located downstream to the I β-catenin-binding repeats, but upstream to the II β-catenin-binding repeats, located between codons 1342 and 2075 (Rubinfeld et al, 1997). Intriguingly, excluding patients with codon 1309 mutation, the highest density of adenomas was observed in one patient with a mutation at codon 1324, that is localized just before the II β-catenin-binding repeats. The possibility that also other mutations localized in this region may associate with a more aggressive phenotype should be further

© 2000 Cancer Research Campaign investigated. In support of this possibility, a previous study showed that mutations localized between codons 1250–1330 (i.e. between the two β-catenin-binding repeats), are associated with a 'profuse' type of polyposis (Nagase et al, 1993).

Regardless of mutation site, the left colon consistently harboured the highest density of adenomas per unit area of mucosal surface, followed by the right colon, sigma, transverse colon and rectum. Regardless of the relative areas of mucosal surface of each colonic segment, the predominance of the absolute number of adenomas in the right colon might account for the prevalently right-sided manifestations of AAPC (Lynch and Smyrk, 1998; Soravia et al, 1998). The degree of phenotypic heterogeneity among patients carrying the same *APC* mutation supported the view that *APC* modifying genes and/or epigenetic and environmental factors may influence the expression of colorectal disease (Borenstein and Dove, 1993; Giardiello et al, 1994; Moser et al, 1995). The colorectal carcinomas, observed in five patients, were all in the left colon, suggesting that regionally prevalent cancer promoting factors might favour left sided adenoma–carcinoma transition.

In conclusion, *APC* mutation sites, although not completely accounting for the intra- and inter-familial variation in the pathophenotype of FAP, provide information that may be relevant for the clinical management of colorectal adenomatosis in FAP carriers. In perspective, surveillance strategies and the timing and extent of prophylactic surgery could be influenced by the specificities of disease manifestations associated with individual APC gene mutations.

### ACKNOWLEDGEMENTS

This work was supported by the Associazione Italiana Ricerca Cancro (AIRC) to AC, Special Project 'Hereditary Colorectal Tumors' and by 40% and 60% grants from the Ministero dell'Universitá e della Ricerca Scientifica e Tecnologica (MURST).

### REFERENCES

Baker RH, Heineman MH, Miller HH and De Cosse JJ (1988) Hyperpigmented lesions of the retinal pigment epithelium in familial adenomatous polyposis. *Am J Med Genet* **31:** 427–435

Bodmer WF, Bailey CJ, Bodmer J, Bussey HJR, Ellis A, Gorman P, Lucibello FC, Murday VA, Rider SH, Scambler P, Sheer D, Solomon E and Spurr NK (1987) Localization of the gene for familial adenomatous polyposis on chromosome 5. *Nature* **328**: 614–616

Borenstein N and Dove W (1993) Genetic identification of Mom-1, a major modifier locus affecting Min-induced intestinal neoplasia in the mouse. *Cell* **75**: 631–639

Cama A, Esposito DL, Palmirotta R, Curia MC, Ranieri A, Ficari F, Valanzano R, Modesti A, Battista P, Tonelli F and Mariani-Costantini R (1994) A novel mutation at the splice junction of exon 9 of the APC gene in familial adenomatous polyposis. *Hum Mutat* **3**: 305–308

Cama A, Sierra ML, Ottini L, Kadowaki T, Imperato-McGinley J and Taylor SI (1991) A mutation in the tyrosine kinase domain of the insulin receptor causing insulin resistance in an obese woman. *J Endocrinol Metab* **73**: 894–899

Cama A, Palmirotta R, Curia MC, Esposito DL, Ranieri A, Ficari F, Valanzano R, Battista P, Modesti A, Tonelli F and Mariani-Costantini R (1995) Multiplex PCR analysis and genotype-phenotype correlations of frequent APC mutations. *Hum Mutat* **5**: 144–152

Caspari R, Friedl W, Mandrel M, Möslein G, Kadmon M, Knapp M, Jacobasch K-H, Ecker KW, Kreiber-Haag D, Timmermanns G and Propping P (1994) Familial adenomatous polyposis: mutation at codon 1309 and early onset of colon cancer. *Lancet* **343**: 629–632

Caspari R, Olschwang S, Friedl W, Mandl M, Boisson C, Boker T, Augustin A, Kadmon M, Möslein G, Thomas G and Propping P (1995) Familial adenomatous polyposis: desmoid tumours and lack of ophthalmic lesions (CHRPE) associated with APC mutations beyond codon 1444. *Hum Mol Genet* **4**: 337–340

Curia MC, Esposito DL, Aceto G, Palmirotta R, Crognale S, Valanzano R, Ficari F, Tonelli F, Battista P, Mariani-Costantini R and Cama A (1998) Transcript dosage effect in familial adenomatous polyposis: model offered by two kindreds with exon 9 APC gene mutations. *Hum Mutat* **11**: 197–201

Dietrich WF, Lander ES, Smith JS, Moser AR, Gould KA, Luongo C, Korinek V, Barker N, Morin PJ, van Wichen D, de Weger R, Kinzler KW, Vogelstein B and Clevers H (1997) Constitutive transcriptional activation by a β-catenin–Tcf complex in $APC^{-/-}$ colon carcinoma. *Science* **275**: 1784–1787

Friedl W, Meuschel S, Caspari R, Lambert C, Krieger S, Sengteller M and Propping P (1996) Attenuated familial adenomatous polyposis due to a mutation in the 3′ part of the APC gene. A clue for understanding the function of the APC protein. *Hum Genet* **97**: 579–584

Gayther SA, Wells D, Sengupta SB, Chapman P, Neale K, Tsioupra K and Delhanty JDA (1994) Regionally clustered APC mutations are associated with severe phenotype and occur at high frequency in new mutation cases of adenomatous polyposis coli. *Hum Mol Genet* **3**: 53–65

Giardiello FM, Krush AJ, Petersen GM, Booker SV, Kerr M, Tong LL and Hamilton SR (1994) Phenotypic variability of familial adenomatous polyposis in 11 unrelated families with identical APC gene mutation. *Gastroenterology* **106**: 1542–1546

Groden J, Thliveris A, Samowitz W, Carlson M, Gelbert L, Albertsen H, Joslyn G, Stevens J, Spirio L, Robertson M, Sargeant L, Krapcho L, Wolff E, Burt R, Hughes JP, Warrington J, Mcpherson J, Wasmuth J, Le Paslier D, Abderrahim H, Cohen D, Leppert M and White R (1991) Identification and characterization of the familial adenomatous polyposis coli gene. *Cell* **66**: 589–600

Groden J, Gelbert L, Thliveris A, Nelson L, Robertson M, Joslyn G, Samowitz W, Spirio L, Carlson M, Burt R, Leppert M and White R (1993) Mutational analysis of patients with adenomatous polyposis: identical inactivating mutations in unrelated individuals. *Am J Hum Genet* **52**: 263–272

Haggitt RC and Reid BJ (1986) Hereditary gastrointestinal polyposis syndromes. *Ann J Surgery Pathol* **10**: 871–887

Jagelman DG (1987) Extracolonic manifestations of familial adenomatous polyposis. *Cancer Genet Cytogenet* **27**: 319–325

Jones IT, Fazio VW, Weakley FL, Jagelman DG, Lavery IC and Megannon E (1986) Desmoid tumors in familial adenomatous polyposis. *Ann Surg* **204**: 94–97

Joslyn G, Richardson DS, White R and Alber T (1993) Dimer formation by an N-terminal coiled-coil in the APC protein. *Proc Natl Acad Sci USA* **90**: 11109–11113

Kinzler KW, Nilbert NC, Su LK, Vogelstein B, Bryan TM, Levy DB, Smith KJ, Preisinger AC, Hedge P, McKechnie D, Finniear R, Markham A, Groffen J, Bogushi MS, Altschul SF, Horii A, Ando H, Miyoshi Y, Miki Y, Nishisho I and Nakamura Y (1991) Identification of FAP locus gene from chromosome 5q21. *Science* **253**: 665–669

Leppert M, Burt R, Hughes J, Samowitz W, Nakamura Y, Woodward S, Gardner E, Lalouel JM and White R (1990) Genetic analysis of an inherited predisposition to colon cancer in a family with a variable number of adenomatous polyps. *N Engl J Med* **322**: 904–908

Lynch HT and Smyrk TC (1998) Classification of familial adenomatous polyposis: a diagnostic nightmare. *Am J Hum Genet* **62**: 1288–1289

Matsumine A, Ogai A, Senda T, Okumura N, Satoh K, Baeg GH, Kawahara T, Kobayashi S, Okada M, Toyoshima K and Akiyama T (1996) Binding of APC to the human homolog of the Drosophila discs large tumor suppressor protein. *Science* **272**: 1020–1023

Miyaki M, Konishi M, Kikuchi-Yanoshita R, Enomoto M, Igari T, Tanaka K, Muraoka M, Takahashi H, Amada Y, Fukayama M, Maeda Y, Iwama T, Mishima Y, Mori T and Koike M (1994) Characteristics of somatic mutations of the adenomatous polyposis gene in colorectal tumors. *Cancer Res* **54**: 3011–3020

Miyoshi Y, Nagase H, Ando H, Horii A, Ichii S, Nakatsuru S, Aoki T, Miki Y, Mori T and Nakamura Y (1992) Somatic mutations of the APC gene in colorectal tumors: mutation cluster region in the APC gene. *Hum Mol Genet* **1**: 229–233

Morin PJ, Sparks AB, Korinek V, Barker N, Clevers H, Vogelstein B and Kinzler KW (1997) Activation of beta-catenin-Tcf signalling in colon cancer by mutations in beta-catenin or APC. *Science* **275**: 1787–1790

Morton DG, Gibson J, MacDonald F, Brown R, Haydon J, Cullen R, Rindl M, Hulten M, Neoptolemos JP, Keighley MRB and McKeown CM (1992) Role of congenital hypertrophy of the retinal pigment epithelium in the predictive diagnosis of familial adenomatous polyposis. *Br J Surg* **79**: 689–693

Moser AR, Luongo C, Gould KA, Mcneley MK, Shoemaker AR and Dove WF (1995) Apc-Min: a mouse model for intestinal and mammary tumorigenesis. *Eur J Cancer* **31A**(7–8): 1061–1064

 © 2000 Cancer Research Campaign

Munemitsu S, Souza B, Müller O, Albert J, Rubinfeld B and Polakis P (1994) The APC gene product associates with microtubules in vivo and promotes their assembly in vitro. *Cancer Res* **54**: 3676–3681

Nagase H and Nakamura Y (1993) Mutations of the APC (adenomatous polyposis coli) gene. *Hum Mutat* **2**: 425–434

Nagase H, Miyoshi H, Horii A, Aoki T, Ogawa M, Utsunomiya J, Baba S, Sasazuki T and Nakamura Y (1992) Correlation between the location of germ-line mutations and the number of colorectal polyps in familial adenomatous polyposis. *Cancer Res* **52**: 4055–4057

Olschwang S, Tiret A, Laurent-Puig P, Muleris M, Parc R and Thomas G (1993) Restriction of ocular fundus lesions to a specific subgroup of APC mutations in adenomatous polyposis coli patients. *Cell* **75**: 959–968

Peifer M, Berg S and Reynolds AB (1994) A repeating amino acid motif shared by proteins with diverse cellular roles. *Cell* **76**: 789–791

Presciuttini S, Varesco L, Sala P, Gismondi V, Rossetti C, Bafico A, Ferrara GB and Bertario L (1994) Age of onset in familial adenomatous polyposis: heterogeneity within families and among APC mutations. *Ann Hum Genet* **58**: 331–334

Rubinfeld B, Souza B, Albert I, Muller O, Chamberlain SH, Masiarz FR, Munemitsu S and Polakis P (1993) Association of the APC gene product with β catenins. *Science* **262**: 1731–1734

Rubinfeld B, Albert I, Porfiri E, Fiol C, Munemitsu S and Polakis P (1996) Binding of GSK3β to the APC-β-catenin complex and regulation of complex assembly. *Science* **272**: 1023–1026

Rubinfeld B, Albert I, Porfiri E, Fiol C, Munemitsu S and Polakis P (1997) Loss of β-catenin regulation by the APC tumor suppressor protein correlates with loss of structure due to common somatic mutations of the gene. *Cancer Res* **57**: 4624–4630

Smith KJ, Johnson KA, Bryan TM, Hill DE, Markowitz S, Willson JK, Paraskeva C, Petersen GM, Hamilton SR, Vogelstein B and Kinzler KW (1993) The APC gene product in normal and tumor cells. *Proc Natl Acad Sci* USA **90**: 2846–2850

Soravia C, Berk T, Madlensky L, Mitri A, Cheng H, Gallinger S, Cohen Z and Bapat B (1998) Genotype–phenotype correlations in attenuated adenomatous polyposis coli. *Am J Hum Genet* **62**: 1290–1301

Sparks AB, Morin PJ, Vogelstein B and Kinzler KW (1998) Mutational analysis of the APC/beta-catenin/Tcf pathway in colorectal cancer. *Cancer Res* **58**: 1130–1134

Spirio L, Olschwang S, Groden J, Robertson M, Samowitz W, Joslyn G, Gelbert L, Thliveris A, Carlson M, Otterud B, Lynch H, Watson P, Lynch P, Laurent-Puig P, Burt R, Hughes JP, Thomas G, Leppert M and White R (1993) Alleles of the APC gene: an attenuated form of familial polyposis. *Cell* **75**: 951–957

Su LK, Vogelstein B and Kinzler KW (1993) Association of the APC tumour suppressor protein with catenins. *Science* **262**: 1734–1737

Utsunomiya J (1989) Pathology, genetics and management of hereditary gastrointestinal polyposis. In: *Genetic Epidemiology of Cancer*, HT Lynch and T Hirayama (eds), pp 219–249

Valanzano R, Cama A, Volpe R, Curia MC, Mencucci R, Palmirotta R, Battista P, Ficari F, Mariani-Costantini R and Tonelli F (1996) Congenital hypertrophy of the retinal pigment epithelium in familial adenomatous polyposis. *Cancer* **78**: 2400–2410

van der Luijt R, Meera Khan P, Vasen H, van Leeuwen C, Tops C, Roest P, den Dunnen J and Fodde R (1994) Rapid detection of translation-terminating mutations at the adenomatous polyposis coli (APC) gene by direct protein truncation test. *Genomics* **20**: 1–4

Yashima K, Nakamori S, Murakami Y, Yamaguchi A, Hayashi K, Ishikawa O, Konishi Y and Sekiya T (1994) Mutations of the adenomatous polyposis coli gene in the mutation cluster region: comparison of human pancreatic and colorectal cancers. *Int J Cancer* **59**: 43–47

*© 2000 Cancer Research Campaign*
